# Optimized workflow for paroxysmal atrial fibrillation ablation using very high power short duration

**DOI:** 10.3389/fcvm.2025.1552340

**Published:** 2025-02-18

**Authors:** Lorenzo Gigli, Alberto Preda, Alessio Testoni, Alexios Sotirios Kotinas, Andrea Tacchetto, Fabrizio Guarracini, Marco Carbonaro, Sara Vargiu, Marisa Varrenti, Giulia Colombo, Roberto Menè, Matteo Baroni, Antonio Frontera, Patrizio Mazzone

**Affiliations:** ^1^ De Gasperis Cardio Center, Electrophysiology Unit, Niguarda Hospital, Milan, Italy; ^2^ Biosense Webster, Johnson & Johnson, Irvine, CA, United States; ^3^ Johnson & Johnson Medical S.p.A, Rome, Italy

**Keywords:** atrial fibrillation ablation, paroxysmal atrial fibrillation, high power short duration, steerable catheter, near zero fluoro ablation

## Abstract

**Background:**

wide antral pulmonary vein isolation (PVI) is effective for treating paroxysmal atrial fibrillation (PAF), although time-demanding. We investigated the impact of a standardized ablation protocol by using a bidirectional transeptal steerable sheath, high-density mapping and very high-power-short-duration (vHPSD) catheters on procedure timing, efficacy, and safety.

**Methods:**

consecutive PAF patients free from previous ablations undergoing PVI alone between January 2022 and March 2023 were prospectively enrolled. The standardized workflow included general anesthesia, a single transeptal puncture trough with a bidirectional, steerable visualizable sheath introduced into the left atrium accommodated a high density, penta-spline mapping catheter and a contact force sensor ablation catheter enabled to deliver vHPSD. Procedural data and electrophysiology (EP) laboratory times were systematically collected and analyzed. The primary endpoint was any AF or atrial tachycardia recurrence at 12 and 24 month follow up.

**Results:**

the study cohort was composed by 138 patients (mean age was 59 ± 11 years, 38% female) and successful PVI was achieved in 100% of cases. Overall, first pass isolation (PFI) was 93%, with a LA dwell time of 32 ± 4 min. Significant complications were reported in 3% of patients. Skin-to-skin time and total EP laboratory time were 58 ± 5 min and 85 ± 7 min, respectively. The primary endpoint was achieved by 9% and 12% of cases at 12 and 24 month follow up, respectively. Upper limit skin-to-skin time and missed FPI resulted predictors of the primary endpoint.

**Conclusion:**

This standardized workflow resulted in low procedural times and arrhythmias recurrence without compromising the safety.

## Highlights

• This is the first study focused on a specific and reproducible step-by-step, systematic workflow for paroxysmal atrial fibrillation ablation using a bidirectional steerable visualizable sheath, high density mapping and vHPSD catheter on a large cohort.
• This workflow achieved very high procedural efficacy as well as long term effectiveness without compromising safety.
• Upper limit skin-to-skin time and missed first pass isolation resulted predictors of arrhythmia recurrence.

## Introduction

Atrial fibrillation (AF) is the most common arrhythmia associated with increased risk of stroke, heart failure and death, hence its prompt treatment and prevention is of utmost importance (1). Pulmonary vein isolation (PVI) is the cornerstone of catheter ablation (CA) and improvements in operator experience and technical advancement have led to reduced complications and favorable long-term arrhythmia-free survival (2). However, since PVI remains a time-demanding procedure, several approaches have been proposed aiming to reduce procedure time without compromise efficacy and safety (3). Data from RCTs and meta-analyses have shown similar data in terms of 1-year freedom from arrhythmia recurrence between point-by-point techniques using radiofrequency (RF) and single-shot ones (4). Novel approaches using high-density mapping catheter and contact force sensor equipped RF catheters enabling temperature-controlled ablation and delivering very high-power short-duration (vHPSD) up to 90 W have provided optimistic results in terms of safety, efficacy and procedure time (5). Since HPSD provides shallower but wider lesions (6), it was specifically developed for posterior PVI, according to less thick left atrial wall, with the aim to reduce collateral tissues damage (7). The association of vHPSD with a bidirectional transeptal visualizable sheath has further optimized the overall workflow by improving quality of lesions and reducing RF time (8, 9). Moreover, performing the procedure by using general anesthesia was related to significant lower rate of AF recurrence compared to sedation in a nationwide cohort (10). In this study, we systematically assessed the implementation of a standardized workflow for paroxysmal AF (PAF) ablation employing high-resolution mapping, vHPSD ablation and a bidirectional transeptal steerable, visualizable sheath.

## Methods

### Study population

We conducted a single-center, prospective study enrolling consecutive patients undergoing first PAF ablation by PVI between March 2021 and August 2022. Inclusion criteria were: >18-years, documented symptomatic PAF and failure of ≥1 antiarrhythmic drug (AAD) (Class I or III). Exclusion criteria were: prior CA of any type, left atrial appendage thrombus evidenced at pre-operatory imaging, need for further ablations other than PVI (i.e., cavo-tricuspid isthmus isolation) and valvular AF. PAF was defined in the presence of at least one AF episode that was continuously sustained less than 7 days, including episodes terminated by cardioversion after <7 days. Patients clinical characteristics, procedural data and follow up were collected and stored in a dedicated dataset. Ethical approval was not required for the studies involving humans because the study is a single-arm observational study using the standard of care. The studies were conducted in accordance with the local legislation and institutional requirements. The participants provided their written informed consent to participate in this study.

### Wide antral circumferential pulmonary vein isolation procedural work-flow

**Figure 1:**
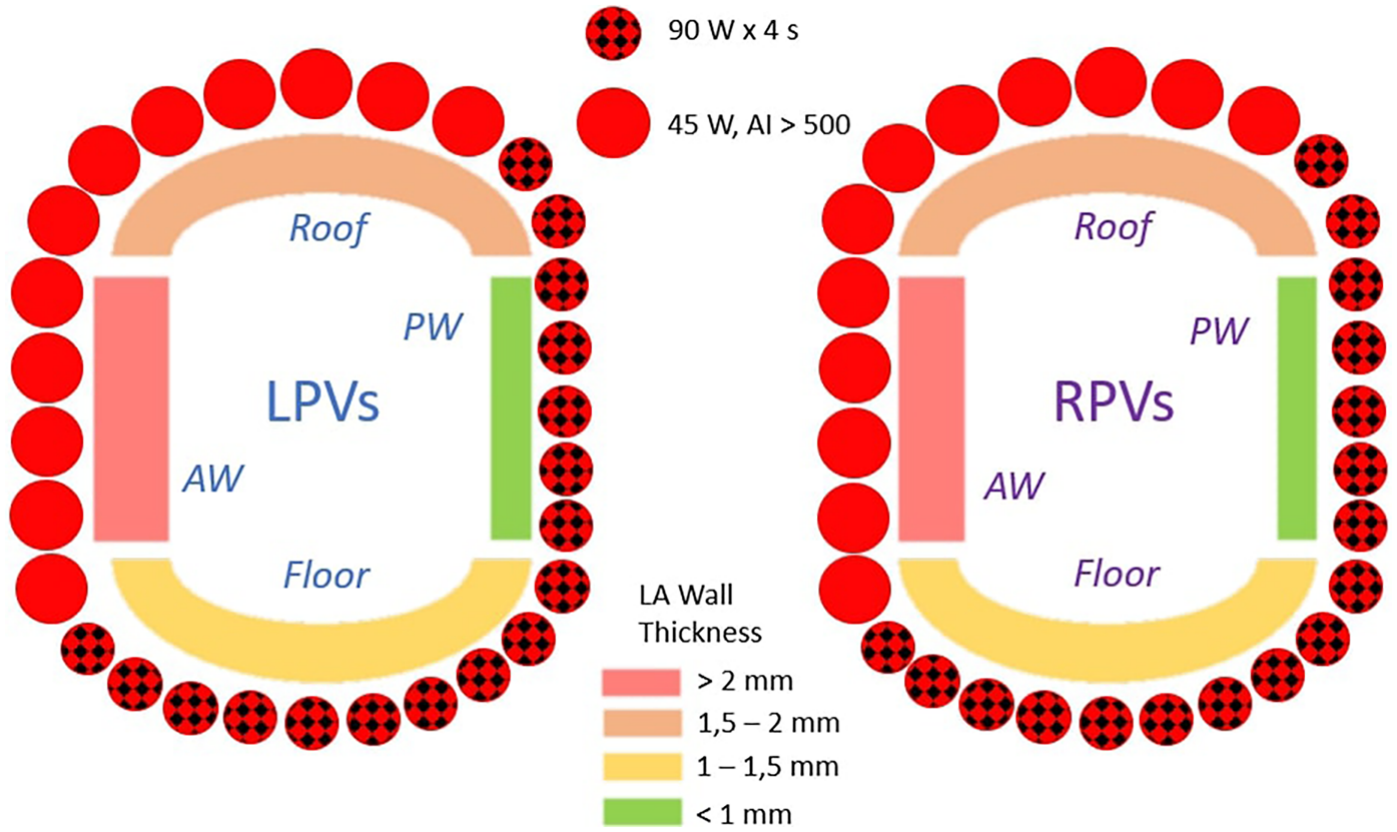
The standard RF protocol based on the average wall thickness of the pulmonary vein antrum. VHSD mode was reserved for the thinner regions, while the conventional Ablation Index guided RF was applied to the thicker regions. AW, anterior wall; LA, left atrium; LPVs, left pulmonary veins, PW, posterior wall; RPVs, right pulmonary veins.

All PVI procedures were guided by the CARTO-3 V7 system (Biosense Webster, USA) under general anesthesia with a conventional ventilation protocol in a volume-controlled mode. Through a triple right femoral venous access, one 9 Fr introducer and two 6 Fr introducers were inserted. Ultrasound (US) guidance was not routinely adopted. A deflectable decapolar catheter was positioned in the coronary sinus through one of the 6 Fr introducers. The remaining 6 Fr introducer was exchanged for the SL0 transseptal sheath Fast-Cath™ (Abbott, USA). Transseptal puncture (TSP) was guided by TOE performed by cardiologists experienced in cardiac imaging and all procedures were performed according to the single catheter approach technique (11). After TSP, a bi-directional, steerable transseptal visualizable sheath (Vizigo™ catheter, Biosense Webster, USA) was introduced into the left atrium (LA) to support both the mapping and the ablation phase. Patients on AF were electrically cardioverted at this step. A penta-splined, high-density mapping catheter (Pentaray™, Biosense Webster, USA) was used to acquire a fast-anatomical map (FAM) of the entire LA anatomy and the pulmonary veins (PVs). Endocardial voltage mapping data were acquired in sinus rhythm by a constant cardiac pacing of 80bpm through medium/distal coronary sinus. Low-voltage areas were defined as areas of endocardial bipolar voltage <0.5 mV and >1 cm^2^ during SR. After the mapping phase, the mapping catheter was positioned into the right atrium through the 9 Fr introducer to better visualize the body of the steerable catheter. The PVI was performed by using a contact force-sensing, thermocouple designed, irrigated ablation catheter enabling very high-energy temperature-controlled ablation reaching 90 W over 4 s (QDOT Micro™ catheter, Biosense Webster, USA). Point-by-point lesions were delivered with a contact force >10 g and a maximal inter-lesion distance of 5 mm. The personalized ablation line was depicted on the 3D bipolar map around the PV antrum according to a wide antral circumferential ablation (WACA) pattern >10 mm outside the PV ostia, where the local electrograms did not show near-field PV signals (12). A RF protocol including vHPSD along the posterior wall and the floor (QMode+™, 90 Watt × 4 s, irrigation flow rate of 8 ml/h),) and a conventional Ablation Index (AI) guided RF (QMode™, 45 Watt × AI 550, irrigation flow rate at 4, increasing to 15 ml/h if measured temperature reached a certain threshold) along the anterior wall and the roof was applied (Figure 1). VHPSD was applied in all cases of carina electrical activity. Acute PVI was confirmed by demonstrating bidirectional block: entry block was demonstrated by the absence of PV potentials inside the vein with the ablation catheter placed sequentially in each segment inside the circumferential PV line and exit block by proving absence of electric capture of the atrium during high-output pacing from inside the circumferential PV line, at each segment sequentially. Additional ablation was delivered at connected sites until PVI was achieved. The procedure was not terminated until confirming the absence of visual gaps between VisiTags. Ventilation and ablation protocols are reported in Table 1. The electrophysiology (EP) laboratory staff consisted of two electrophysiologists, one cardiology specialized in procedural echocardiography, one anesthesiologist and two nurses. Ablation was carried out by four different first operators with a median range of invasive experience of 9 years (range 6–27 years). Heparin was administered targeting an activated clotting time >300 s. In the case of direct oral anticoagulant therapy, medication was withheld on the day of the procedure and restarted 6 h after catheter ablation. Major complications including periprocedural death, atrioesophageal fistula, periprocedural thromboembolic event, cardiac tamponade, vascular complication with the need of surgical or percutaneous intervention were reported.

**Table 1:** Protocol for standard high-frequency low-tidal volume ventilation.

FiO2 (%)	30
Ventilation rate (breath/min)	12–13
Inspiration/expiration ratio	1–2
Tidal volume (ml/kg)	6–8
Radiofrequency protocol	
Anterior wall and roof	45 W × AI > 520
Posterior wall and floor	90 W × 4 s
Carina	45 W × AI > 520

### Workflow analysis of peri-procedural care and intra-procedural measurements

sing a specific computer program (Pace Insights, Biosense Webster), peri- and intra-procedural steps were documented starting when the patient entered the EP laboratory. Four procedural steps (pre-procedural preparation, vascular access and transseptal puncture, LA mapping and ablation) were recorded. Total procedure time and skin-to-skin time from vascular access to closure were assessed. Further analysis included the number and duration of RF applications.

### Post-procedural care and follow up

Femoral access sites were closed by a figure-of-eight suture and manual compression. Pericardial effusion was excluded by transthoracic echocardiography (TTE) after the procedure. AAD therapy was prescribed on an individual basis for each patient. All patients have been recovered for at least one night after ablation. After discharge, patients were scheduled for follow up at the outpatient clinic at 3, 6, 12 and 24 months after the procedure. Each evaluation included an ECG and 24 h Holter ECG monitoring. The primary outcome was freedom from sustained atrial arrhythmia recurrence after a blanking period of 3 months, defined as any documented AF, atrial flutter, or atrial tachycardia (AT) episode lasting more than 30 s, regardless of symptoms. AADs withdraw was left to the medical choice.

### Statistical analysis

Continuous variables were presented as mean ± standard deviation or median (interquartile range), as appropriate, based on the Shapiro-Wilk test to assess the normality of the data distribution. Categorical variables were presented as counts (%). Kaplan–Meier curves and the log-rank test were used to assess cumulative AF/AT-free survival. Logistic regression models were performed to identify predictors of AF/AT recurrence during the follow-up. A level of *p* < 0.05 was considered for statistical significance. Data were analyzed with R version 3.6.2 software (R Foundation for Statistical Computing, Vienna, Austria).

## Results

### Baseline population

A total of 138 consecutive patients (mean age was 64.5 ± 9.5 years, 31% female) meeting the inclusion criteria were enrolled during the study period. Baseline clinical characteristics are summarized in Table 2. Hypertension and dyslipidemia were the most frequent risk factors. Mean indexed LA volume was 37 ± 9, corresponding to a mild dilation. A cardiomyopathy was reported in 24 (17%) patients, being the idiopathic dilated type the most frequent one, and the left ventricle (LV) function was impaired in 32 patients (23%). The mean time between AF diagnosis and the procedure was 11.8 ± 5.7 months. Almost all patients were on double AAD at the moment of admission, being beta-blocker and flecainide and the most frequent association. At the moment of the in-hospital admission, 13 patients (9%) reported ongoing AF.

**Table 2:** Patients baseline characteristics.

N. of patients	138
Male	86 (62%)
Age, years	59 ± 11
Concomitant clinical conditions	
Hypertension	78 (57%)
Diabetes mellitus	27 (20%)
Dyslipidaemia	40 (29%)
Obesity	21 (15%)
Smoke history	18 (13%)
CKD	9 (7%)
Coronary artery disease	15 (11%)
Previous ischemic cerebral events	5 (4%)
Obstructive sleep apnoea syndrome	9 (7%)
Cardiomyopathy	24 (17%)
CIED	5 (4%)
History of cardiac surgery	3 (2%)
LVEF >50%	106 (77%)
LAVi, ml/BSA	37 ± 9
MR > moderate	6 (4%)
CHA2-DS2-VASc Score	3 ± 1
Time since AF diagnosis, months	11.8 ± 5.7
Longest AF episode, days	4 ± 2
AAD at baseline – no. (%)	
Beta-blocker	102 (74%)
Flecainide	74 (54%)
Propafenone	22 (16%)
Sotalol	7 (5%)
Amiodarone	24 (17%)

AAD, antiarrhythmic drug; AF, atrial fibrillation; CIED, cardiac implantable electronic device; CKD, chronic kidney disease; MR, mitral regurgitation; LAVi, left atrial volume index; LVEF, left ventricular ejection fraction.

### Workflow analysis

**Figure 2:**
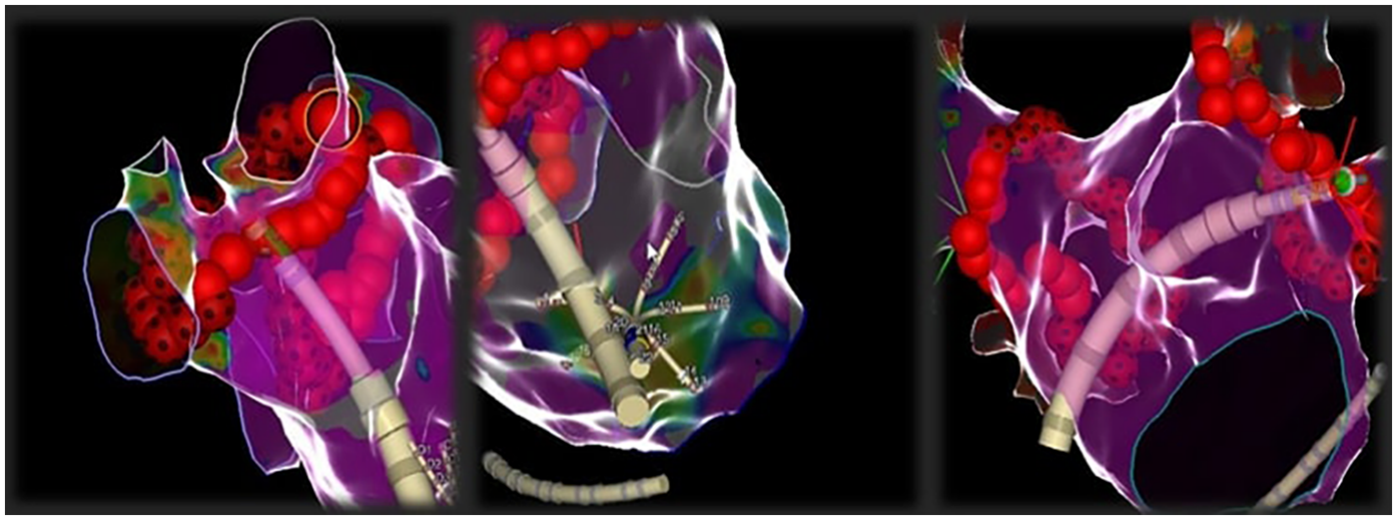
Representative images of electroanatomical mapping during ablation. During the ablation phase, the mapping catheter is positioned in the right atrium to allow the visualization of the steerable catheter along its entire length into the left atrium.

The procedural data and the results of the EP laboratory interval analysis are shown in Table 3. Acute PVI was achieved in all procedures, with a FPI of 93% and a mean LA dwell time of 32 ± 4 min. Major complications occurred in four patients (3%): one intraprocedural severe pericardial effusion related to the transseptal puncture requiring urgent pericardiocentesis and three femoral access complications requiring percutaneous treatment. No patients experienced pericarditis or esophagus injury. No neurological complications were observed. The mean total laboratory time and skin-to-skin time were 85 ± 7 min and 58 ± 5 min, respectively. The FAM acquisition time was 13 ± 2 min. The fluoroscopy time was 61 ± 3 s. Overall, left atrial dwell time was 32 ± 4 min. LA mapping revealed LA low-voltage areas in 9 (7%) patients. The mean number of VisiTags was 74 ± 6, of which 69 ± 4 for the left pulmonary veins and 77 ± 5 for the right pulmonary veins, including the carinas. The mean RF time was 18 ± 3 min. Figure 2 shows the electroanatomical map including the equipment set up during the procedure.

**Table 3:** Procedural data.

AF at the beginning of the procedure	12 (9%)
Preprocedural preparation, min	15 ± 3
Procedure time skin-to-skin, min	58 ± 5
FAM time, min	13 ± 2
Fluoroscopy time, s	61 ± 3
Total VisiTags	74 ± 6
Low-voltage area > 1 cm^2^ during SR	9 (7%)
Total RF time, min	17 ± 3
Right PVs	10 ± 2
Left PVs	7 ± 2
LA dwell time, min	32 ± 4
Mean anterior AI	556 ± 7
Carina electrical activity	8 (6%)
Right PV	5 (4%)
Left PV	3 (2%)
Pulmonary veins first-pass isolation	
Right PVs first-pass isolation	126 (91%)
Left PVs first-pass isolation	128 (93%)
Major procedural complication	4 (3%)
Cardiac tamponade	1 (0.7%)
Femoral access complications requiring percutaneous treatment	3 (2%)
Postprocedural preparation, min	12 ± 3
Total laboratory time, min	85 ± 7

AF, atrial fibrillation; AI, ablation index; LA, left atrium; PV, pulmonary vein; RF, radiofrequency; SR, sinus rhythm.

### Clinical outcomes

**Figure 3:**
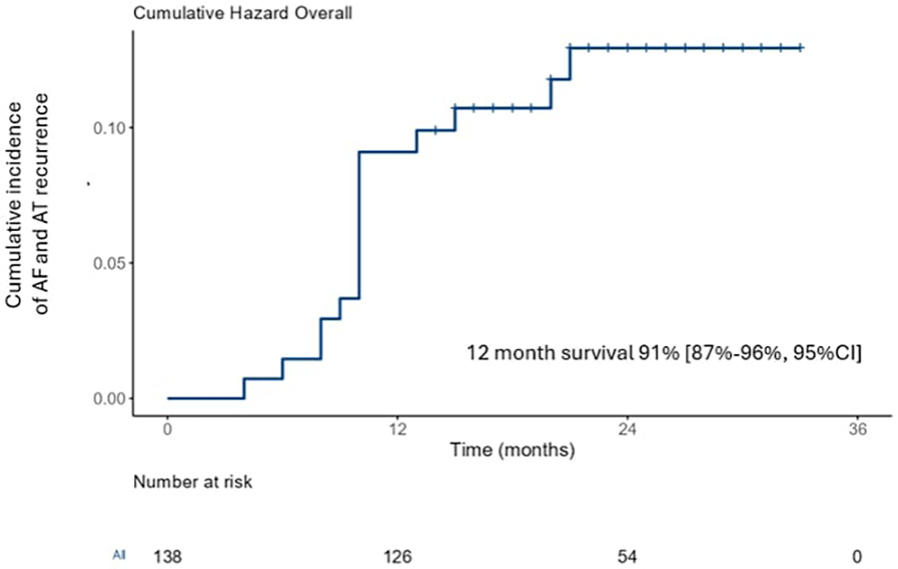
Kaplan Meyer curve of cumulative incidence of the primary endpoint at follow up.

The mean follow-up was 29 ± 4 months. No deaths were reported. All patients were maintained on AADs during the blanking period (3 months) and 15 patients (11%) continued taking AADs after its end. At 12-month and 24-month follow up, 12 patients (91%) and 16 patients (12%) achieved the primary outcome (Figure 3). The major part experienced AF recurrence (14, 88%) while only 2 cases reported atypical atrial flutter. Of them, 11 patients underwent REDO ablation and 5 patients (45%) showed PVs reconnection. At univariate and multivariate analysis (Table 4), upper limits skin-to-skin times [1.22 (95%CI 1.08–1.98, *p* = 0.041)] and missed FPI [1.08 (95%CI 1.02–1.56, *p* = 0.046)] resulted predictors of primary outcome.

**Table 4:** Univariate and multivariate analysis of primary endpoint occurrence.

Parameter	Univariate OR (95% CI)	*p*-value	Multivariate OR (95% CI)	*p*-value
Age, years	1.04 (0.95–2.11)	0.816		
Obesity	1.23 (0.91–2.31)	0.253		
LVEF <50%	1.69 (0.84–2.22)	0.068		
Time since AF diagnosis, months	1.84 (0.56–4.32)	0.658		
AF at the beginning of the procedure	1.13 (0.95–1.58)	0.075		
Skin-to-skin time, min	1.34 (1.06–1.86)	0.036	1.22 (1.08–1.98)	0.041
AAD withdrawal within 1-year	1.57 (0.93–2.87)	0.138		
Missed first-pass isolation	1.10 (1.04–1.87)	0.041	1.08 (1.02–1.56)	0.046

AAD, antiarrhythmic drug; AF, atrial fibrillation; LVEF; left ventricle ejection fraction; PVI, pulmonary vein isolation.

## Discussion

This is a the first study focused on a specific and reproducible step-by-step, systematic workflow for PAF ablation on a wide cohort of PVI patients under general anesthesia and a combined RF protocol according to the LA wall thickness. All procedural phases in the EP laboratory were collected. The main results of our study were: (i) high EP laboratory efficacy, (ii) low overall LA dwell time, (iii) high FPI, and (iv) very low arrhythmia recurrence at long term follow up, with 75% of recurrence within 1 year from the procedure.

### Contact force sensing vHPSD ablation equipped with bidirectional steerable visualizable sheath

According to several studies, vHPSD ablation provides effective applications with very low risk of collateral damage and decreased RF time for PVI (13–15), achieving significant time saving compared to conventional RF as well as radiation dosage and fluoroscopy exposure (5, 16). Despite EP laboratory time was not systematically assessed in all studies, improvements in the overall operator experience as well as in the working processes may also have been concurred to the reduction of the procedure time (17). Lower effectiveness of 90W application compared to AI guided 45 W has been reported, according to the differences in lesion characteristics and the higher sensitivity of the vHPSD to catheter instability (18). This is the reason why our protocol provided a combined (90–45 W) according to the LA wall thickness associated to the use of a transeptal steerable sheath. Indeed, vHPSD applications may be well suited for the posterior wall of the LA, which is less thick compared to the anterior one (2–3 mm vs. up to 6 mm), other than in the proximity of the esophagus (19). This balance between the effectiveness and safety was the rationale for the site-specific power settings of our strategy. This strategy, in addition to the use of general anesthesia, were the two key factors differentiating our approach from the most similar one recently published by Fink et al., which employed vHPSD along both the anterior and posterior PV ostium (5). Moreover, the integration of the bidirectional visualizable sheath contributed to remarkable improvements regarding procedure duration and radiation exposure, hereby further enhancing the efficacy of PVI by increased catheter stability during RF applications (20, 21). The adoption of these techniques may explain the reason to such increase in FPI reported in our study compared with recent ones using a similar workflow (22, 23). VHPSD on LA posterior wall and floor was safe and effective in our study. All patients with electrically active carina underwent conventional 45W ablation because persistent connections are known common cause of failed FPI (24). The primary outcome at 12 months was 91%, being among the highest reported in literature, and was maintained at 24-month follow up. Of note, some risk factors related to AF/AT recurrence were identified. Longer procedure times were probably related to more complex PVs anatomy leading to unsatisfactory contact during RF application while missed FPI confirmed itself as a well known risk factors for recurrence (25). In our study, only 11% of patients continued AAD therapy three months after AF ablation, following the recommendation of the specialist at the time. Recent guidelines do not provide any specific recommendations on this topic. At our center, this decision is based on the duration of the arrhythmia in the patient's history, the number of recurrences while on antiarrhythmic therapy, the size of the left atrium, and the patient's age.

### A systematic, step by step optimized workflow for high-efficacy, safety and time-saving AF ablation

Given the increasing number of AF ablations performed annually worldwide and the economic constraints in healthcare systems, a highly effective and timely procedure is a crucial component of routine workflows for interventional procedures today. Our approach is characterized by a single TSP through which a high-density mapping catheter and a novel ablation catheter equipped with a force sensor and capable of delivering vHPSD are introduced in the LA through a bidirectional steerable visualizable sheath. This resulted in significant laboratory time saving without compromising the efficacy and safety. The combination of high-density mapping with vHPSD ablation represents the most advanced setup for point-to-point AF ablation available to date, providing superior results compared to other point-to-point approaches in terms of procedure time, acute and long-term safety and efficacy, as reported in recent single-center and multicenter studies (5, 15). Accordingly, our systematic analysis showed that the two main drivers of procedure time shortening were decreased mapping and ablation times, with subsequently low fluoroscopy time. However, only few studies reported a systematic analysis of EP laboratory time intervals (5). We excluded patients who were indicated for cavo-tricuspid isthmus isolation to avoid potential bias that could influence procedure times. Systematic assessment of laboratory time intervals can serve as an important tool for the optimization of procedural workflows, improving both acute and long-term outcomes as well as overall costs (26, 27). The use of a standardized stepwise near-zero fluoroscopy approach with TOE-guided TSP is currently the gold standard and contributes to further reductions in fluoroscopy and complications (28). According to the workflow, US guidance for femoral venous access was not routinely adopted, despite US has been shown to effectively reduce both major and minor complications by 3–4 times, as reported in recent studies and meta-analyses (29, 30). In accordance with these studies, the rate of vascular complications in our cohort could have been reduced from 2% to 1%. Fluoroscopy has always been the cornerstone imaging method in electrophysiology, even with the advent of 3D mapping systems (31). However, radiation exposure is associated with an increased risk of malignancies and multi-organ diseases. In our study, fluoroscopy was almost entirely limited to the TSP, achieving a mean time of one minute, which is 3–4 times lower than what has been reported in previous studies on the same topic. In conclusion, procedure time remains a critical factor, as higher complication rates have generally been observed in cases with longer procedures, regardless of the strategy used (32).

### Advanced point-to-point radiofrequency ablation in the era of single-shot techniques

RF is currently the main source of energy delivered during catheter-based PVI and it is now used to achieve ablations with continuous, point-by-point application of thermal injury around the PV antrum. However, this approach has been considered by many electrophysiologists as time-consuming, at high-risk for procedural complications, and suboptimal for achieving durable PVI. Given the increasing elderly population with drug refractory PAF, research has focused on the development of faster and safer technologies. The cryoablation, introduced over a decade later, can achieved PVI with a single circumferential ablation lesion, demonstrating similar results to RF in the most recent trials in terms of long-term effectiveness [around 35% arrhythmia recurrence at 1.5-years follow up in Fire and Ice trial (33) and around 47%–49% at 1-year follow up in CIRCA-DOSE trial] and complications (10 vs.12%, *p* = 0.24 in Fire and Ice and 2.5%–5%, *p* = 0.31 in CIRCA-DOSE). On the other hand, significantly shorter procedure time was achieved by cryoablation compared to RF (124–130 min vs. 140–160 min, respectively). More recently, advancements in expertise and technology have led to a reduction in arrhythmia recurrence to as low as 25% at 1 year in patients treated with this technique (34). The novel pulsed field ablation (PFA) is rapidly spreading due to its reduced procedure times and low rate of complications (35–37). Nevertheless, the most recent and largest studies report a 1-year freedom from AF recurrence rate of between 66% and 75% in patients with PAF, while data for follow-up beyond 1 year are currently lacking (35, 38, 39). Recent improvement in RF catheters equipped with contact-force sensors enabling HPSD ablation, in the context of a standardized procedural workflow, have reported very high efficacy (around 87%–90% at 1-year), low complication rate (<5%) and reduced procedure times (around 60–65 min) compared to the past (15, 40). Our study reinforces the findings of previous research by demonstrating the effects of a systematic procedural workflow on a large cohort, confirming the effectiveness of this technique over a two-year period.

### Limitations

This is a prospective, non-randomized single center study with its typical limitations. The major limitation is the lack of a control group, making it more challenging to determine whether the outcome was caused by the experimental treatment or by other variables. Finally, intermittent rhythm monitoring with ECGs and Holter studies can underestimate arrhythmia burden post-PVI.

## Conclusion

In the era of single-shot techniques, RF applied with an optimized procedural protocol has demonstrated low procedural times, low rate of complications, and increased long-term effectiveness, still maintaining a notable role in the treatment of PAF.

## Data Availability

The raw data supporting the conclusions of this article will be made available by the authors, without undue reservation.
